# Rifampin use in acute community-acquired meningitis in intensive care units: the French retrospective cohort ACAM-ICU study

**DOI:** 10.1186/s13054-015-1021-7

**Published:** 2015-08-26

**Authors:** Cédric Bretonnière, Mathieu Jozwiak, Christophe Girault, Pascal Beuret, Jean-Louis Trouillet, Nadia Anguel, Jocelyne Caillon, Gilles Potel, Daniel Villers, David Boutoille, Christophe Guitton

**Affiliations:** Service de Réanimation Médicale Polyvalente, CHU Nantes, Pôle Hospitalo-universitaire 3, place A. Ricordeau, Nantes, F-44093 France; Faculté de Médecine, Université de Nantes, UPRES EA 3826, 1 rue Gaston Veil, Nantes, F-44035 France; Service de Réanimation Médicale, AP-HP, Hôpitaux universitaires Paris-Sud, Hôpital de Bicêtre, 78 rue du Général Leclerc, Le Kremlin-Bicêtre, F-94270 France; Service de Réanimation Médicale Polyvalente, CHU-Hôpitaux de Rouen, Hôpital Charles Nicolle, 1 rue de Germont, Rouen, F-76000 France; Groupe de Recherche sur le Handicap Ventilatoire (GRHV), UPRES EA 3830-Institut de Recherche et d’Innovation Biomédicale (IRIB), Faculté de Médecine et de Pharmacie, Université de Rouen, Rouen, F-76031 France; CH Roanne, Service de Réanimation Médico-chirurgicale, 28 rue de Charlieu, Roanne, F-42300 France; Service de Réanimation Médicale, AP-HP, Hôpital de la Pitié-Salpêtrière, Institut de Cardiologie, 47-53 boulevard de l’Hôpital, Paris, F-75651 France; Service de Bactériologie, CHU Nantes, Pôle Hospitalo-universitaire 7, place A. Ricordeau, Nantes, F-44093 France; Service d’Accueil des Urgences, CHU Nantes, Pôle Hospitalo-universitaire 3, ’place A. Ricordeau, Nantes, F-44093 France; Service des Maladies Infectieuses, CHU Nantes, Pôle Hospitalo-universitaire 3, place A. Ricordeau, Nantes, F-44093 France

## Abstract

**Introduction:**

Bacterial meningitis among critically ill adult patients remains associated with both high mortality and frequent, persistent disability. Vancomycin was added to treatment with a third-generation cephalosporin as recommended by French national guidelines. Because animal model studies had suggested interest in the use of rifampin for treatment of bacterial meningitis, and after the introduction of early corticosteroid therapy (in 2002), there was a trend toward increasing rifampin use for intensive care unit (ICU) patients. The aim of this article is to report on this practice.

**Methods:**

Five ICUs participated in the study. Baseline characteristics and treatment data were retrospectively collected from charts of patients admitted with a diagnosis of acute bacterial meningitis during a 5-year period (2004–2008). The ICU mortality was the main outcome measure; Glasgow Outcome Scale and 3-month mortality were also assessed.

**Results:**

One hundred fifty-seven patients were included. *Streptococcus pneumoniae* and *Neisseria meningitidis* were the most prevalent causative microorganisms. The ICU mortality rate was 15 %. High doses of a cephalosporin were the most prevalent initial antimicrobial treatment. The delay between admission and administration of the first antibiotic dose was correlated with ICU mortality. Rifampin was used with a cephalosporin for 32 patients (ranging from 8 % of the cohort for 2004 to 30 % in 2008). Administration of rifampin within the first 24 h of hospitalization could be associated with a lower ICU survival. Statistical association between such an early rifampin treatment and ICU mortality reached significance only for patients with pneumococcal meningitis (*p*=0.031) in univariate analysis, but not in the logistic model.

**Conclusions:**

We report on the role of rifampin use for patients with community-acquired meningitis, and the results of this study suggest that this practice may be associated with lower mortality in the ICU. Nevertheless, the only independent predictors of ICU mortality were organ failure and pneumococcal infection. Further studies are required to confirm these results and to explain how rifampin use would reduce mortality.

**Electronic supplementary material:**

The online version of this article (doi:10.1186/s13054-015-1021-7) contains supplementary material, which is available to authorized users.

## Introduction

Bacterial meningitis among adult patients remains associated with both high mortality and frequent, persistent disability, especially in critically ill patients. Early antimicrobial treatment is the cornerstone of the management of these infections [1–8]. Coadministration of corticosteroids has made major progress in the last decade. Indeed, in 2002, de Gans and van de Beek demonstrated that early administration of dexamethasone improves both mortality and neurological outcome [9]. However, such a use of corticosteroids yielded many questions [8, 10]. What about antibiotic diffusion into the cerebrospinal fluid (CSF)? Would it be decreased?

French guidelines added a second antibiotic to a cephalosporin for those patients at risk of contracting a penicillin-resistant or less susceptible strain or for clinically more severe infections [11]. Therefore, patients admitted to the intensive care unit (ICU) were generally treated with a third-generation cephalosporin and vancomycin. After widespread steroid use, French physicians wondered whether the efficacy of vancomycin would be maintained [12, 13]. At the same time, other antibiotics, such as fosfomycin and rifampin, were shown to have pharmacokinetic and pharmacodynamic properties that could make them good candidates for the treatment of meningeal infections [14]. Using rifampin appeared at that time to be an interesting concept because previous experimental studies in animal models had suggested that rifampin reduces the inflammatory response caused by β-lactam–induced bacterial lysis [15, 16].

The aim of this report is to describe a cohort of adult patients admitted to the ICU for community-acquired bacterial meningitis, with a specific focus on rifampin use.

## Methods

### Study design/center characteristics

This study was multicentered and retrospective. Five French ICUs participated. Four of them are teaching medical ICUs: Nantes, Rouen, Paris, and Kremlin-Bicêtre. The fifth, Roanne, is a general medicosurgical ICU. During the study period, these ICUs had, respectively, 20, 20, 18, 15, and 16 ventilated beds. Yearly admissions to the ICU during the study period ranged from 650 to 1100.

### Inclusion criteria

All sequential patients admitted to the ICUs were assessed for inclusion in the study. Adult patients were included if they met the following criteria:

- Admission to one of the five participating ICUs during the 5-year period from 1 January 2004 through 31 December 2008
- Admission for community-acquired bacterial meningitis

The patients’ clinical presentation had to be consistent with this diagnosis. CSF samples had to show pleiocytosis (white cell count >5/mm^3^). In those patients with an obvious clinicobiological diagnosis but a contraindication to lumbar puncture (e.g., major coagulation abnormalities), missing CSF analysis was permitted.

Exclusion criteria were nosocomial meningitis, tuberculosis diagnosis, viral or parasitic etiology, cerebral abscess, or secondary localizations of endocarditis.

### Data collection

#### Patients’ baseline characteristics

The following data were taken from charts: demographic data, comorbidities, clinical characteristics at emergency department and ICU admission, and severity measures such as the Simplified Acute Physiology Score II (SAPS II) [17] or the Logistic Organ Dysfunction (LOD) system [18].

#### Outcome

The main outcome assessment was ICU mortality, but 3-month mortality and Glasgow Outcome Scale (GOS) score were also recorded when possible. The GOS is widely used to assess disability after severe brain damage or after medical conditions such as meningeal infections. In brief, it has five categories: dead, vegetative, severely disabled, moderately disabled, and good recovery [19].

#### Microbiological data

CSF characteristics were analyzed. Bacterial identification could also be derived from other samples, such as blood cultures or skin cultures.

Antimicrobial susceptibility was tested according to French national guidelines [20] and in accordance with the European Committee on Antimicrobial Susceptibility Testing. Thus, minimal inhibitory concentrations (MICs) for *Streptococcus pneumoniae* were as follows:

1. *Penicillin*: susceptible, MIC ≤0.06 mg/ml; intermediate, MIC 0.1–1 mg/ml; and resistant, MIC >1 mg/ml
2. *Amoxicillin*: susceptible, MIC ≤0.5 mg/ml; intermediate, MIC 1–2 mg/ml; and resistant, MIC >2 mg/ml
3. *Third-generation cephalosporins (cefotaxime or ceftriaxone)*: susceptible, MIC ≤0.5 mg/ml; intermediate, MIC 1–2 mg/ml; and resistant, MIC >2 mg/ml

#### Treatment data

Antibiotic regimens were described according to type of antibiotics, doses, eventual combination, and timing of administration. Steroid administration was also investigated. Data concerning antibiotic tolerance were also documented from patients’ charts. Considered adverse events (AEs) were those that were sufficiently serious for clinicians to be mentioned in the ICU hospitalization report.

### Statistical analysis

Results were expressed as mean ± standard deviation or median with interquartile range (IQR). The categorical data were presented as percentages. These data were compared with the Fisher’s exact test, and continuous data were compared with Student’s *t* test when normality criteria (Kolmogorov-Smirnov test) were fulfilled or with non-parametric tests in case of non-normality. Missing data were treated in the simplest approach: analyses were performed on only the completed cases. Missing data were excluded from the analysis.

The main outcome measure was ICU mortality. To investigate the link between different variables and ICU mortality, a binary logistic regression model was used. Variables with a *p* value <0.20 in univariate analysis were entered into the models. The final model was kept because of its ability to best predict the outcome, maximizing the likelihood ratio. The highest Nagelkerke’s *R*^2^ then obtained is presented with the final model. Use of a Cox model would have been an option. However, follow-up times were highly variable. This made us prefer a logistic regression model.

Analyses with a *p* value <0.05 were considered statistically significant. The analysis was carried out using IBM SPSS Statistics version 19 software (IBM, Armonk, NY, USA).

### Ethical approval

The protocol was approved by the ethics committee of the French Intensive Care Society, Société de Réanimation de Langue Française. Because of the retrospective nature of this study and according to French and European laws, no informed consent was required.

## Results

More than 20,000 patients were admitted to the 5 participating ICUs during the 5-year study period. One hundred fifty-seven patients (1.2 % of the ICU admissions) were included in this study. The patients’ mean age was 45±20 years. Ninety-seven (62 %) were men. The following predisposing conditions were found: alcohol use disorders (n=18; 11 %), previous episode of meningitis (n=8; 5 %), HIV infection (n=8; 5 %), and asplenia (n=6; 4 %). At the ICU admission, the mean Glasgow Coma Scale score was 11±4, and the mean SAPS II was 33±21. Assessed in the LOD system, the neurologic system was the most frequently involved organ dysfunction at ICU admission (60 %), whereas pulmonary, hematologic, cardiovascular, and renal failures were present in 44 %, 42 %, 37 %, and 24 % of the cases, respectively. No patient had liver failure. At admission, the criteria for severe sepsis or septic shock were fulfilled for 84 % of the patients. Mechanical ventilation was needed for 44 % of them.

Median length of ICU stay was 4 days (IQR 2–7). ICU mortality was 15 %, and 3-month mortality was 22 %. GOS scores were available for 120 patients with a median follow-up period of 231 days. Three months after admission, 24 patients were dead (GOS 1), none were in a persistent vegetative state (GOS 2), and 4 had severe disability (GOS 3). Ninety patients (76 %) were considered to have a favorable outcome: 7 had moderate disability (GOS 4), and 85 had a good recovery (GOS 5).

No difference was observed between centers (no center effect for demographic data, microbiological data, or treatment).

### Microbiological data

Lumbar puncture was contraindicated in four patients owing to coagulation disorders. For 30 of 153 patients, CSF culture was sterile. Seven of those had a positive microscopic result. Microbiological data were also available from blood (n=25) or skin cultures (n=2) or from molecular techniques (PCR on CSF; n=21). Overall, microbiological documentation was available for 136 patients (87 %). The main causative bacteria were *S. pneumoniae* (56 %) and *Neisseria meningitidis* (32 %). The results are shown in Additional file 1: Figure S1. Among *S. pneumoniae* strains, susceptibility to β-lactams was as follows: (1) intermediate or resistant to penicillin, 48 %; (2) intermediate or resistant to amoxicillin, 11 %; and (3) intermediate or resistant to third-generation cephalosporins, 7 %. The rate of penicillin non-susceptible pneumococcal strains was 60 % in 2004. That rate dropped to 40 % in 2006 and remained around that level until 2008.

### Therapeutic measures

**Fig. 1:**
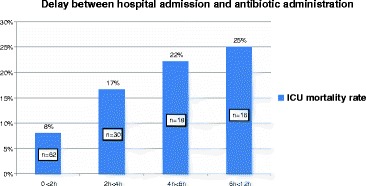
Correlation between time of antibiotic administration and intensive care unit (ICU) mortality for patients admitted for bacterial meningitis

**Fig. 2:**
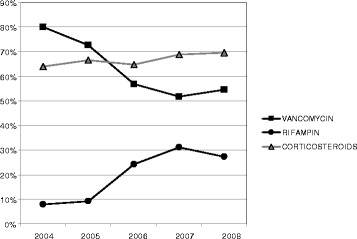
The proportion of patients treated with vancomycin, rifampin, and corticosteroids between 2004 and 2008

The mean time between hospital admission and first antibiotic administration was 2.5 h (IQR 1.0-6.1). There was lower ICU mortality for patients with earlier antibiotic treatment (Fig. 1). Among ICU survivors, median time (IQR) between hospital admission and first antibiotic administration was 1.6 (0.6–3.7) versus 3.8 (2.1–5.5) for non-survivors (*p*=0.003). An antibiotic combination was the initial prescription for 119 patients (76 %). A third-generation cephalosporin (89 %) was generally used with vancomycin (68 %) or rifampin (23 %). During the study period, vancomycin use decreased and rifampin use became more frequent. Corticosteroid therapy was administered to 105 patients (67 %). The treatment consisted of a conventional treatment with dexamethasone for 91 patients and low-dose hydrocortisone for septic shock for 12 others. Two received another regimen. The rate of steroid therapy was stable during the study period. Figure 2 summarizes these results.

For pneumococcal infections, an initial combination of antibiotics was more frequently used (95 % vs. 58 %; *p*<0.0001). Corticosteroids were also more frequently administered (82 % vs. 53 %; *p*<0.001).

### Rifampin use

Among 157 patients, 32 (20 %) received rifampin. Rifampin use was equally distributed in all five ICUs. It was never prescribed as a monotherapy, but always in addition to a third-generation cephalosporin. It was administered within the first 24 h of hospitalization for 19 patients (59 %), between 24 and 48 h for 6 patients (19 %), and after 48 h for 7 (22 %) of them. In the 76 patients with a final diagnosis of pneumococcal meningitis, 20 (26 %) were treated with rifampin. Two-thirds (n=13) of them received rifampin within the first 24 h of hospital admission.

Patient demographics, severity at ICU admission, and microbiological data did not differ between the patients who did or did not receive rifampin. Corticosteroid therapy was used more frequently in those who received rifampin. A detailed comparison of these groups is shown in Table 1.

**Table 1:** Comparison of patient characteristics, severity at ICU admission, microbiological data, and therapeutic measures between patients treated with rifampin or not for community-acquired bacterial meningitis (n=157)

Characteristics	Rifampin		No rifampin		*p* Value
	n=32		n=125		
Demographic data		IQR		IQR	
Age, yr	41.4	[28.7–59.4]	51.1	[34.6–69.3]	0.087
Female sex	12/32	37.5 %	48/125	38.4 %	NS
Immunosuppression	6/32	18.8 %	23/125	18.4 %	NS
Alcohol use disorder	4/31	12.9 %	14/124	11.3 %	NS
Asplenia	1/32	3.1 %	5/124	4.0 %	NS
HIV infection	1/32	3.1 %	7/124	5.6 %	NS
Severity at ICU admission					
Glasgow Coma Scale score	11	[8–14]	12	[7–15]	NS
Mechanical ventilation	11/25	44.0 %	49/111	44.1 %	NS
Severe sepsis or septic shock	21/25	84.0 %	94/111	84.7 %	NS
SAPS II	29	[16–43]	30	[20–48]	NS
LOD system					
Neurologic	14/25	56.0 %	68/111	61.3 %	NS
Pulmonary	11/25	44.0 %	49/111	44.1 %	NS
Hematologic	11/25	44.0 %	46/111	41.4 %	NS
Cardiovascular	12/25	48.0 %	38/111	34.2 %	NS
Renal	5/25	20.0 %	27/111	24.3 %	NS
Hepatic	–		–		
Microbiology					
CSF white cell count/mm^3^	1710	[450–5100]	1820	[315–3300]	NS
CSF protein rate, g/L	3.8	[2–5.9]	2.7	[1.5–5.8]	NS
Positive direct examination of CSF	21/32	65.6 %	80/120	66.7 %	NS
*Streptococcus pneumoniae* as causative bacterium	20/32	62.5 %	56/125	44.8 %	0.079
Penicillin non-susceptible isolates	11/20	55,0 %	21/46	45,7 %	NS
*Neisseria meningitidis* as causative bacterium	5/32	15.6 %	39/125	31.2 %	0.081
Therapeutic measures					
Time between hospital admission and first dose of antibiotic (h)	2.5	[0.8–6.1]	3.2	[1.5–6.9]	NS
Treatment with corticosteroids during hospitalization	28/32	87.5 %	77/125	61.6 %	**0.006**

*SAPS II* Simplified Acute Physiology Score II, *CSF* cerebrospinal fluid, *LOD* Logistic Organ Dysfunction system, *NS* not significant

Data are proportion of patients or median values [interquartile range]

*p* Values <0.2 are detailed. *p* Values <0.05 were considered significant (bold)

Rifampin was administered twice daily. Doses were either 600 mg or 900 mg twice daily, depending on the patient’s weight (about 10 mg/kg twice daily).

In the entire population (n=157), none of the patients treated with early rifampin (within the first 24 h of hospitalization) died. The difference in mortality reached statistical significance only for patients with pneumococcal infection. Indeed, among the latter 76 patients, none of the 18 non-survivors were treated with early rifampin (during the first 24 h of hospitalization), whereas 13 of 58 patients did receive early rifampin among the survivors (*p*=0.031) (Table 2).

**Table 2:** Correlation between ICU mortality and rifampin treatment in critically ill patients admitted with bacterial meningitis

Rifampin use	Non-survivors		Survivors		*p* Value
Entire population	n=23		n=134		
Rifampin during hospitalization	2/23	8.7 %	30/134	22.4 %	NS
Rifampin during first 48 h of hospitalization	1/23	4.3 %	23/134	17.2 %	NS
Rifampin during first 24 h of hospitalization	0/23	0.0 %	19/134	14.2 %	0.078
*Streptococcus pneumoniae* meningitis	n=18		n=58		
Rifampin during hospitalization	2/18	11.1 %	18/58	31.0 %	NS
Rifampin during first 48 h of hospitalization	1/18	5.6 %	15/58	25.9 %	0.097
Rifampin during first 24 h of hospitalization	0/18	0.0 %	13/58	22.4 %	**0.031**

*NS* not significant

Data are proportions of patients. They were compared with Fisher’s exact test

*p* Values <0.1 are detailed. *p* Values <0.05 were considered significant (bold)

The GOS was also statistically associated with the use of rifampin, with the rifampin-treated patients having a statistically better GOS score than the others (Additional file 1: Figures S2 and S3).

For the entire cohort, 10 antibiotic-related AEs were reported (6 %). Two events were associated not with antibiotics prescribed for the initial bacterial meningitis, but with another prescription during the hospitalization. Two AEs were related to vancomycin use (inflammation at injection site). Six were reported with the use of a β-lactam: abnormal liver tests (n=2), encephalopathy (n=1), *Clostridium difficile* diarrhea (n=1), and oral candidiasis (n=1). No AEs were reported for rifampin use.

### Mortality-associated factors

Factors associated with ICU mortality were analyzed for the whole population. The results of the univariate analysis are summarized in Table 3. On the basis of the results of univariate analyzes, we aimed to build different logistic regression models in order to more accurately assess whether treatment with rifampin was associated with outcome. Demographic parameters, data on severity at admission, and treatment data such as antibiotics and corticosteroids were entered into the model. Possible interactions between variables were also tested. The variables were alternately added or removed from the model to test how they acted on this model. Those variables were selected that allowed the highest likelihood ratio. For the final logistic analysis, 11 variables were kept in the model: pulmonary, renal, cardiovascular, and hematologic failure; pneumococcal infection; early antibiotic administration (<2 h) and early treatment with rifampin (<24 h); corticosteroid administration; age (>75 years) and sex; and immunosuppression. The results are presented in Table 4. Specific results for patients with pneumococcal meningitis are shown in Additional file 1: Tables S1 and S2).

**Table 3:** Comparison of patient characteristics, severity at ICU admission, microbiological data, and therapeutic measures between survivors and non-survivors after ICU admission for bacterial meningitis (n=157)

Characteristics	Non-survivors		Survivors		*p* Value
	n=23		n=134		
Demographics		IQR		IQR	
Age, yr	65	[40.6–74.3]	41	[28.7–59.1]	**0.003**
Female sex	4/23	17.4 %	56/134	41.8 %	**0.035**
Immunosuppression	9/23	39.1 %	20/134	14.9 %	**0.016**
Alcohol use disorder	7/23	30.4 %	11/132	8.3 %	**0.007**
Asplenia	2/23	8.7 %	4/133	3.0 %	NS
HIV infection	3/23	13.0 %	5/133	3.8 %	0.096
Severity at ICU admission					
Glasgow Coma Scale score	7	[4–9]	12	[9–15]	**<10** ^**−3**^
Mechanical ventilation	20/21	95.2 %	40/115	34.8 %	**0.004**
Severe sepsis or septic shock	21/21	100.0 %	94/115	81.7 %	**0.043**
SAPS II	64	[53–73]	27	[15–36]	**<10** ^**−3**^
LOD system					
Neurologic	21/21	100.0 %	61/115	53.0 %	**<10** ^**−3**^
Pulmonary	20/21	95.2 %	40/115	34.8 %	**<10** ^**−3**^
Hematologic	12/21	57.1 %	45/115	39.1 %	0.151
Cardiovascular	12/21	57.1 %	38/115	33.0 %	**0.048**
Renal	13/21	61.9 %	19/115	16.5 %	**<10** ^**−3**^
Hepatic	0/21	0.0 %	0/115	0.0 %	–
Microbiology					
CSF white cell count/mm^3^	1900	[290–4500]	1770	[494–5050]	NS
CSF protein rate, g/L	8.4	[4.7–10.6]	3.1	[1.8–4.9]	**<10** ^**−3**^
Positive direct examination of CSF	17/22	77.3 %	84/130	64.6 %	NS
*Streptococcus pneumoniae* as causative bacterium	18/23	81.8 %	58/134	43.3 %	**0.003**
Penicillin non-susceptible isolates	7/14	31.8 %	25/52	48.1 %	NS
*Neisseria meningitidis* as causative bacterium	1/23	4.5 %	43/134	32.1 %	**0.005**
Therapeutic measures					
Time between hospital admission and first dose of antibiotic (h)	4	[1.9–8.5]	2.4	[0.7–6]	0.102
Association of antibiotics as initial therapy	22/23	95.7 %	97/134	72.4 %	**0.016**
Treatment with rifampin during the hospitalization	2/23	8.7 %	30/134	22.4 %	0.168
Early treatment with rifampin (within first 24 h)	0/23	0.0 %	19/134	14.2 %	0.078
Treatment with vancomycin during hospitalization	20/23	87.0 %	78/134	58.2 %	**0.01**
Early treatment with vancomycin (within first 24 h)	17/23	73.9 %	66/134	49.3 %	**0.041**
Treatment with corticosteroids during hospitalization	18/23	78.3 %	87/134	64.9 %	NS
Time between hospital admission and first dose of corticosteroids (h)	8	[5–13]	5.4	[1.7–9.5]	**0.042**

*SAPS II* Simplified Acute Physiology Score II, *CSF* cerebrospinal fluid, *LOD* Logistic Organ Dysfunction system

Data are proportions of patients and percentages or median values [interquartile range]

*p* Values <0.2 are detailed. *p* Values <0.05 were considered significant (bold)

**Table 4:** Factors associated with ICU mortality in a cohort of 157 patients admitted for bacterial meningitis

Variable	Odds ratio (95 % CI)	*p* Value
Pulmonary failure	67.7 (3.7–1225.7)	**0.004**
Renal failure	12.6 (2.2–73.1)	**0.005**
Cardiovascular failure	9.8 (1.7–57.6)	**0.012**
Pneumococcal infection	9.0 (1.1–74.8)	**0.042**
Early antibiotic administration (<2 h)	0.3 (0.0–1.6)	0.155
Hematologic failure	3.6 (0.6–20.6)	0.156
Corticosteroid administration	4.1 (0.5–36.2)	0.200
Age (>75 yr)		NS
Immunosuppression		NS
Female sex		NS
Early treatment with rifampin (<24 h)		NS

Incomplete observations were excluded from the logistic analysis. The presented final model had 134 observations entered and yielded the highest likelihood ratio. Nagelkerke’s *R* ^2^ was 0.69. The 11 presented variables were those included in the final logistic model

*p* Values <0.2 are detailed. *p* Values <0.05 were considered significant (bold)

## Discussion

In this report, we describe a cohort of 157 patients. Meningeal infections are rare conditions. So, in the present study, for five ICUs and during the study period, these infections represented only 1.2 % of admissions (among more than 20,000 admitted patients).

Patients’ baseline characteristics are coherent in terms of severity. Some might be surprised by the high Glasgow Coma Scale scores (median 11) or low SAPS II, but these data are consistent with those reported in other series [2, 4]. We must emphasize that, at admission, 80 % of the patients met criteria for severe sepsis or septic shock and that for nearly half of them mechanical ventilation was required. A previous study included patients with more severe illness. They had exclusively pneumococcal meningitis [1].

From a microbiological point of view, our results are not surprising. The two main causative bacteria are *S. pneumoniae* and *N. meningitidis*. This predominance is typical of what is reported in the literature [5].

The originality of this work lies in the analysis of rifampin use to treat bacterial meningitis. As discussed in the Introduction, the years 2002–2005 appeared to be marked by a change in prescriptions of antibiotics for bacterial meningitis in France. Our study confirms this characterization. Thus, vancomycin prescriptions were very common in 2004 and then fell sharply. The rise of rifampin lies in the potentially very interesting properties of the molecule. This drug has been known for a long time [21]. It is widely used for tuberculosis and bone or joint staphylococcal infections [22, 23]. It can also be used as chemoprophylaxis in close contacts with patients with meningococcal meningitis. It indeed has very interesting pharmacokinetic properties, particularly its cerebromeningeal distribution. This is facilitated by its lipophilic properties and its low molecular weight [24, 25]. In addition, for *S. pneumoniae*, MICs to rifampin are extremely low [26]. Resistance remains exceptional [27]. Moreover, unlike vancomycin, its distribution is unaffected by the addition of corticosteroid therapy [10]. Based on these attractive pharmacokinetic and pharmacodynamic properties, animal studies have demonstrated the relevance of this molecule for curative treatment of meningitis [15, 28–31]. It could have a protective effect in limiting inflammatory mediator release [32–37]. Reactive oxygen species and matrix metalloproteases have both been shown to contribute to brain damage [38–40]. Their production could be limited by using non β-lactam antibiotics [41]. For all these reasons and despite the lack of robust data, clinicians have chosen to use this rifampin. Given precisely the lack of data in the literature, it is interesting to report on this clinical experience. However, our preliminary data do not specify for which patients this treatment would be the most beneficial. Our clinical view and preclinical data suggest that, if effective, this treatment should be administered early (in the very first hours) and in patients with pneumococcal infection.

Our study also clarifies the factors associated with ICU mortality. Thus, pneumococcal infections are again identified as being associated with a worse outcome [1]. Our study shows that the patients with most severe illness at admission have the highest probability of death, which is classic [42]. Shock is thus an independent risk factor of mortality. In addition, we can specify that the presence at admission of a respiratory or renal dysfunction weighs heavily on the outcome. Neurological impairment at admission is so common in this cohort that it does not emerge as significantly associated with mortality in the multivariate analysis. In contrast to previous studies, delay in the first antibiotic administration or pneumococcal phenotypes does not appear to be independently associated with mortality [43–45]. Precocity of antibiotic administration is indeed a cornerstone of the treatment for these severe infections. Although they did not reach statistical significance, our results confirm this fact. Concerning non-susceptibility to penicillin, it had been associated with mortality in a previous French study [45]. The latter could weigh in favor of rifampin use because, even for penicillin non-susceptible pneumococcal strains, MICs remain low for this antibiotic. Our results might differ because of the higher prevalence of penicillin non-susceptible *S. pneumoniae* (50 %) in comparison with the cited study (36 %).

However, our work has many limitations. The first is that it included a small number of patients. Although the cohort had a significant size, only 20 % of them (n =32) were treated with rifampin. The number was even smaller in the subgroup that received early treatment (within the very first hours). However, this use of rifampin has not been reported previously in humans. In addition, new recommendations issued in late 2008 put an end to the use of an antibiotic combination for bacterial meningitis and promoted high doses of cephalosporin as monotherapy [46]. It was then not possible to extend the study period.

The main limitation of the present study is its retrospective characteristic. We are indeed not able to explain why clinicians have or have not chosen to administer rifampin. Although we did not show significant major differences between patients with or without rifampin, the former more frequently received corticosteroids. This may have played a role in the superiority of rifampin versus no rifampin observed in the univariate analysis.

Nevertheless, we must not overestimate our results. Whereas our univariate analysis shows a signal in favor of a potential interest of rifampin use, multivariate analysis showed no association between ICU mortality and rifampin use. Indeed, this study might be underpowered related to the small size of the rifampin-treated patient subgroup (n=32). It is true that multivariate analysis does not show either link with corticosteroid use. However, for steroids in this study, it should be noted that they were often administered as published by de Gans in 2002 [9] but sometimes differently. For instance, some patients with septic shock have received low doses of hydrocortisone instead of dexamethasone and hydrocortisone on metabolic parameters (e.g., glucose and glucose variability).

The use of rifampin may also be criticized. Rapid emergence of resistant strains requires its use only in combination therapy [22]. Although our study showed good tolerance, this molecule can lead to significant AEs [47]. Drug interactions require the cautious use of multiple therapies [48].

The interest in an association could be discussed. As mentioned before, local recommendations in France changed in late 2009. These actually stress the need for a monotherapy with high doses of a third-generation cephalosporin. However, owing to the versatile epidemiology of pneumococcal infections and the mortality associated with bacterial meningitis, new potential therapeutic data seem welcome.

## Conclusions

Our study presents a cohort of adult ICU patients and specifically focuses on the use of rifampin in combination with a third-generation cephalosporin to treat community-acquired bacterial meningitis. This treatment could be of interest for patients with *S. pneumoniae* infection, especially if administered early. Nevertheless, these results are preliminary and can pave the way for further studies.

## Key messages

- Rifampin in combination with a third-generation cephalosporin is an option to treat community-acquired bacterial meningitis.
- Such a treatment may reduce mortality in the ICU (univariate analysis).
- Our study demonstrates that only four independent factors were associated with ICU mortality: pulmonary failure, renal failure, cardiovascular failure, and pneumococcal infection.

## Additional file

Additional file 1: Figure S1. Bacteria involved in 136 episodes of meningitis. **Figure S2.** Glasgow Outcome Scale scores for the groups of patients who did or did not receive rifampin for community-acquired meningitis. **Figure S3.** Glasgow Outcome Scale scores for the groups of patients who did or not receive early rifampin (within the first 24 h of hospitalization) or not for community-acquired meningitis. **Table S1.** Comparison of patient characteristics, severity at ICU admission, 9pt?>microbiological data, and therapeutic measures between survivors and non-survivors after ICU admission for pneumococcal meningitis (n=76). **Table S2.** Factors associated with ICU mortality for a cohort of 76 patients admitted for pneumococcal meningitis. (PDF 157 kb)

## Abbreviations

AE: Adverse event
CSF: Cerebrospinal fluid
GOS: Glasgow Outcome Scale
ICU: Intensive care unit
IQR: Interquartile range
LOD: Logistic Organ Dysfunction
MIC: Minimal inhibitory concentration
SAPS II: Simplified Acute Physiology Score II
