# Supplementary material for: Rifampin use in acute community-acquired meningitis in intensive care units: the French retrospective cohort ACAM-ICU study
Source: Crit Care. 2015 Aug 26;19(1):303. doi: 10.1186/s13054-015-1021-7 (PMC4549935; doi:10.1186/s13054-015-1021-7)
Supplement: Additional file 1: Figure S1. — Bacteria involved in 136 episodes of meningitis. Figure S2. Glasgow Outcome Scale scores for the groups of patients who did or did not receive rifampin for community-acquired meningitis. Figure S3. Glasgow Outcome Scale scores for the groups of patients who did or not receive early rifampin (within the first 24 h of hospitalization) or not for community-acquired meningitis. Table S1. Comparison of patient characteristics, severity at ICU admission, 9pt?>microbiological data, and therapeutic measures between survivors and non-survivors after ICU admission for pneumococcal meningitis (n=76). Table S2. Factors associated with ICU mortality for a cohort of 76 patients admitted for pneumococcal meningitis. (PDF 157 kb) [file 13054_2015_1021_MOESM1_ESM.pdf]

Supplementary data

Figures

Figure S1: Bacteria involved in 136 episodes of meningitis

Figure S2: Glasgow Outcome Scale for the groups of patients who did or did not receive rifampin for a community acquired meningitis (n=157)

Figure S3: Glasgow Outcome Scale for the groups of patients who did or not receive early rifampin (within the 24 first hours of hospitalization) or not for a community acquired meningitis (n=157)

Tables

Table S1: Comparison of patient characteristics, severity at ICU admission, microbiological data and therapeutic measures between survivors and non survivors after ICU admission for pneumococcal meningitis (n=76)

Table S2: Factors associated with ICU mortality for a cohort of 76 patients admitted for pneumococcal meningitis

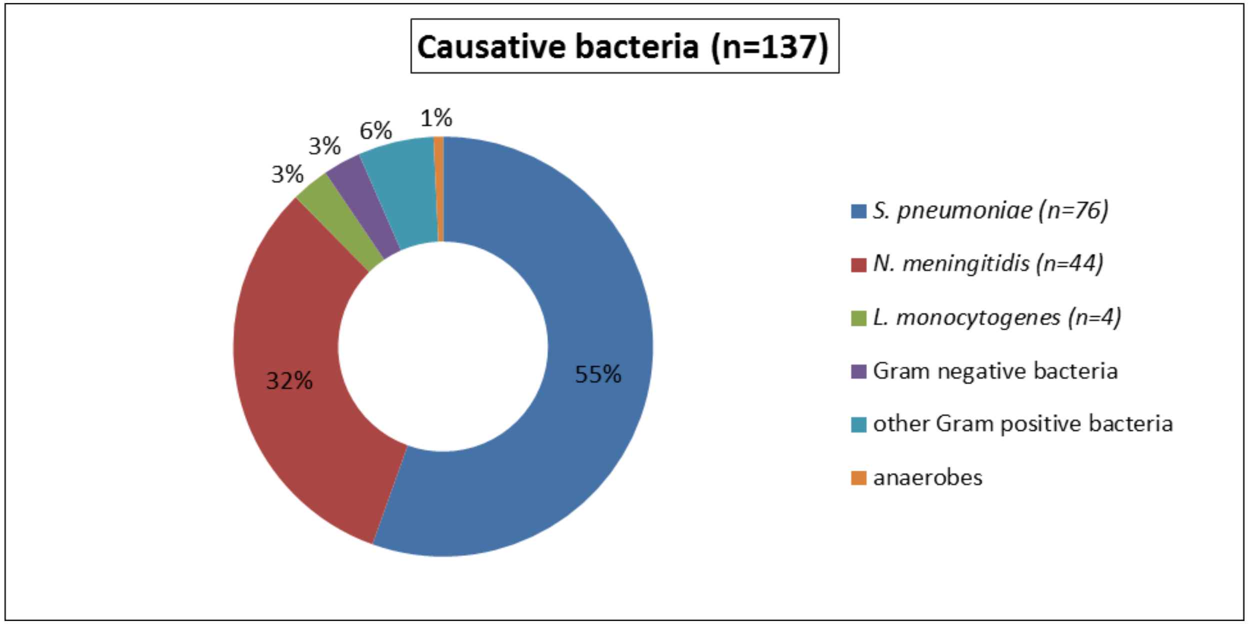

Figure S1: Bacteria involved in 136 episodes of meningitis

One patient had a co-infection so that 137 bacteria were involved in 136 infections.

Gram negative bacteria (n=4): *Haemophilus influenzae* (n=2), *Klebsiella pneumoniae* (n=1), *Capnocytophaga canimorsus* (n=1)

Other Gram positive bacteria (n=7): *Staphylococcus aureus* (n=2), *Streptococcus pyogenes* (n=2), *Streptococcus agalactiae* (n=2), *Streptococcus salivarius* (n=1), *Streptococcus intermedius* (n=1)

Anaerobes (n=1): *Fusobacterium sp.* (n=1; co-infection with *Streptococcus intermedius*)

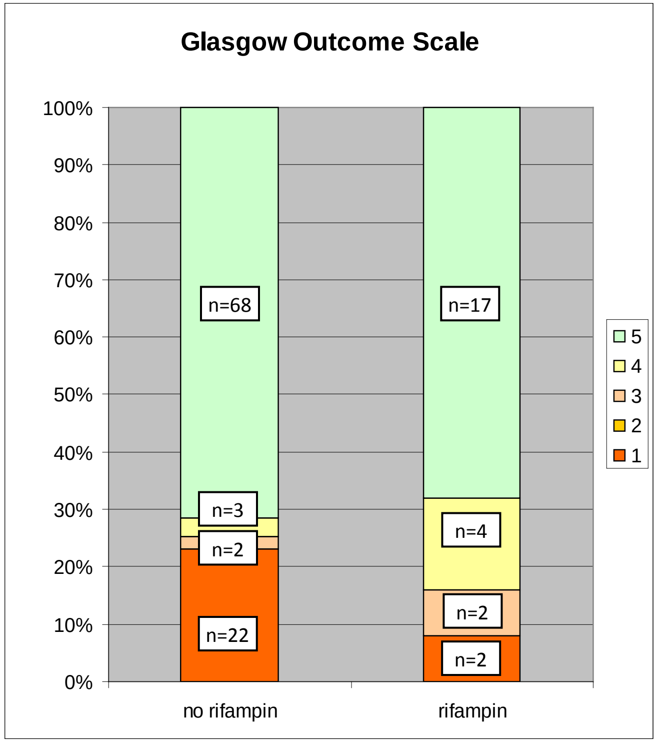

Figure S2: Glasgow Outcome Scale for the groups of patients who did or did not receive rifampin for a community-acquired meningitis

GOS at day 90 was available for 120 patients. None of them had a score of 2 (persistent vegetative state). Fisher's test showed statistical difference for GOS between the 2 subgroups of patients who had received or not rifampin *(p=0.017)*

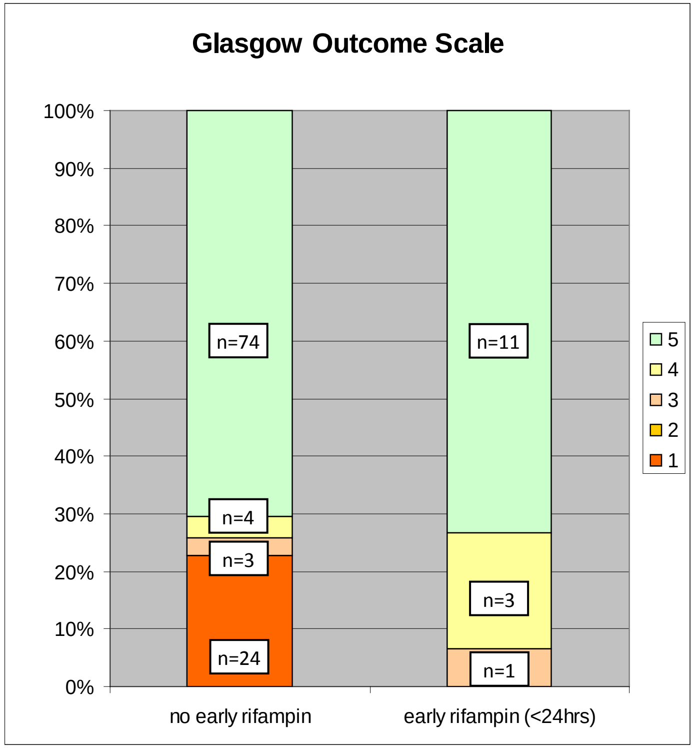

Figure S3: Glasgow Outcome Scale for the groups of patients who did or not receive early rifampin (within the 24 first hours of hospitalization) or not for a community-acquired meningitis

GOS at day 90 was available for 120 patients. None of them had a score of 2 (persistent vegetative state) and no patient had a score of 1 in the *'early rifampin'* group.
Fisher's exact test showed statistical difference for GOS between the 2 subgroups of patients who had received early rifampin or no early rifampin *(p=0.014)*

	Non survivors n=18		Survivors n=58		*p-value*
**Patient characteristics**					
Age	67.2	*[43.1 74.5]*	49.7	*[38.3 63.8]*	*0.124*
Female sex	3/18	*16,7%*	25/58	*43,1%*	*0.053*
Immunosuppression	6/18	*33,3%*	15/58	*25,9%*	*NS*
Alcohol use disorder	4/18	*22,2%*	9/56	*16,1%*	*NS*
Asplenia	1/18	*5,6%*	3/58	*5,2%*	*NS*
HIV infection	2/18	*11,1%*	3/57	*5,3%*	*NS*
**Severity at ICU admission**					
Glasgow Coma Scale	6	*[3 7]*	10	*[8 13]*	***<0.001***
SAPS2	65	*[53 77]*	33	*[24 44]*	***<0.001***
Depressed conscious level	17/17	*100,0%*	34/45	*75,6%*	***0.026***
Respiratory failure	16/17	*94,1%*	24/45	*53,3%*	***0.003***
Hematological failure	9/17	*52,9%*	24/45	*53,3%*	*NS*
Cardiovascular failure	10/17	*58,8%*	13/45	*28,9%*	***0.041***
Renal failure	9/17	*52,9%*	8/45	*17,8%*	***0.01***
Hepatic failure	-		-		
**Microbiology**					
CSF white-cell count per mm3	1500	*[290 3100]*	1740	*[388 5300]*	*NS*
CSF protein rate g/L	8.7	*[5.1 10.7]*	3.2	*[2.3 5.9]*	***0.001***
Positive direct examination of CSF	13/17	*76,5%*	48/58	*82,8%*	*NS*
**Therapeutic measures**					
Time between hospital admission and first dose of antibiotic (hours)	3.8	*[1.5 7]*	2.5	*1 7*	*NS*
Combination of antibiotics as initial therapy	17/18	*94,4%*	55/58	*94,8%*	*NS*
Treatment with rifampin during the hospitalization	2/18	*11,1%*	18/58	*31,0%*	*0.129*
Early treatment with rifampin (within the 24 first hours)	0/18	*0,0%*	13/58	*22,4%*	***0.031***
Treatment with vancomycin during the hospitalization	15/18	*83,3%*	47/58	*81,0%*	*NS*
Early treatment with vancomycin (within the 24 first hours)	12/18	*66,7%*	40/58	*69,0%*	*NS*
Treatment with corticosteroids during the hospitalization	14/18	*77,8%*	48/58	*82,8%*	*NS*
Time between hospital admission and first dose of corticosteroids (hours)	8	*[5.6 13]*	5.6	*[1.6 7.5]*	***0.034***

Table S1: Comparison of patient characteristics, severity at ICU admission, microbiological data and therapeutic measures between survivors and non survivors after ICU admission for pneumococcal meningitis (n=76)

Data are proportion (%) of patients or median values *[interquartile range]*.
P-value<.2 are detailed. P-value<.05 were considered significant (bold).

	OR	*95% IC*		*p*
Cardiovascular failure	7.5	*[1.3*	*44.2]*	***0.027***
Renal failure	7.5	*[1.3*	*44.2]*	***0.027***
Respiratory failure	21.6	*[1.2*	*404.5]*	***0.040***
Early antibiotics administration (<2hrs)				*NS*
Hematological failure				*NS*
Age (>75)				*NS*
Corticosteroids administration				*NS*
Female sex				*NS*
Immunosuppression				*NS*
Early treatment with rifampin (<24hrs)				*NS*

Table S2: Factors associated with ICU mortality for a cohort of 76 patients admitted for pneumococcal meningitis

Incomplete observations were excluded from the binary logistic analysis. The presented model is from 62 observations and yielded the highest likelihood ratio.
P-value<.2 are detailed. P-value<.05 were considered significant (bold).
